# A review of the response and the emergency medical team (EMT) deployment following a tanker explosion in Freetown, Sierra Leone

**DOI:** 10.1186/s13031-024-00619-z

**Published:** 2024-10-21

**Authors:** Boniface Oyugi, Ibrahim Franklyn Kamara, Innocent Nuwagira, Robert Musoke, Sulaiman Lakoh, Abdulai Jalloh, Rashidatu Fouad Kamara, Pryanka Relan, Camila Lajolo, René André Macodou Ndiaye, Babacar Niang, Mouhamadou Mansour Fall, Thierno Balde, Flavio Salio, Mustapha Kabba

**Affiliations:** 1 World Health Organisation, Emergency Medical Teams Initiative, Headquarters, Geneva, Switzerland; 2 Centre for Health Services Studies (CHSS), University of Kent, George Allen Wing, Canterbury, CT2 7NF UK; 3 World Health Organisation, Country Office, Freetown, Sierra Leone; 4 Ministry of Health and Sanitation, Freetown, Sierra Leone; 5 World Health Organisation, Emergency Preparedness and Response Programme,, Regional Office for Africa, Brazzaville, Congo; 6 Hôpital Principal de Dakar, Dakar, Senegal; 7 World Health Organisation, Emergency Preparedness and Response Programme,, Regional Hub for West Africa, Dakar, Senegal

**Keywords:** Sierra Leone, Deployment, Tanker explosion, Response, Emergency medical teams

## Abstract

**Background:**

On 5 November 2021, a fire incident following a tanker explosion occurred in the Wellington PMB Junction east of Freetown, Sierra Leone, injuring and killing people. WHO facilitated the deployment of international emergency medical teams (EMTs) to support the Ministry of Health (MoH) in providing care to the wounded in four hospitals.

**Objective:**

In this study, we document Sierra Leone's experience managing the fire incident and the role of EMTs in responding to it.

**Method:**

This is a cross-sectional After-Action Review (AAR) debrief of the response and deployment, including focus group discussion with WHO and MoH staff (n = 14) in a virtual workshop and document reviews on the response. The results thematically cover the event and the different agencies' responses and a review of EMTs' responses.

**Results:**

At the onset of the emergency, the National Disaster Management Agency (NDMA) instituted a well-coordinated response mechanism in collaboration with the MoH and managed all response actions, such as medical services, informing partners and the public and coordinating all other agencies. WHO facilitated EMT deployments and mobilised medical supplies and equipment, while the MoH provided accommodation, logistics and coordination. The EMTs dispensed their functions with professionalism, adapted to the environment and available resources, and augmented the care the national health workers provided. They offered additional care: reconstructive surgery, pain management, palliative care, wound care, rehabilitation, physiotherapy and psychosocial counselling, which were initially inadequate at the onset of the disaster. 94 out of 157 patients were discharged home at the end. National clinicians acquired additional skills through the capacity-building activities of EMTs. The community appreciated the teams.

**Conclusion:**

The government, partners and EMTs were important in the response and worked with speed and political acceptability using the context experience to provide surge support to the country. This experience brought to focus the idea of developing a national EMT in Sierra Leone, which would be useful to help respond even more swiftly. In collaboration with WHO, there is a need to institute further mechanisms to facilitate rapid response and quality-assured deployment of EMTs at regional and sub-regional levels and strengthen to support future responses.

## Background

**Fig. 1:**
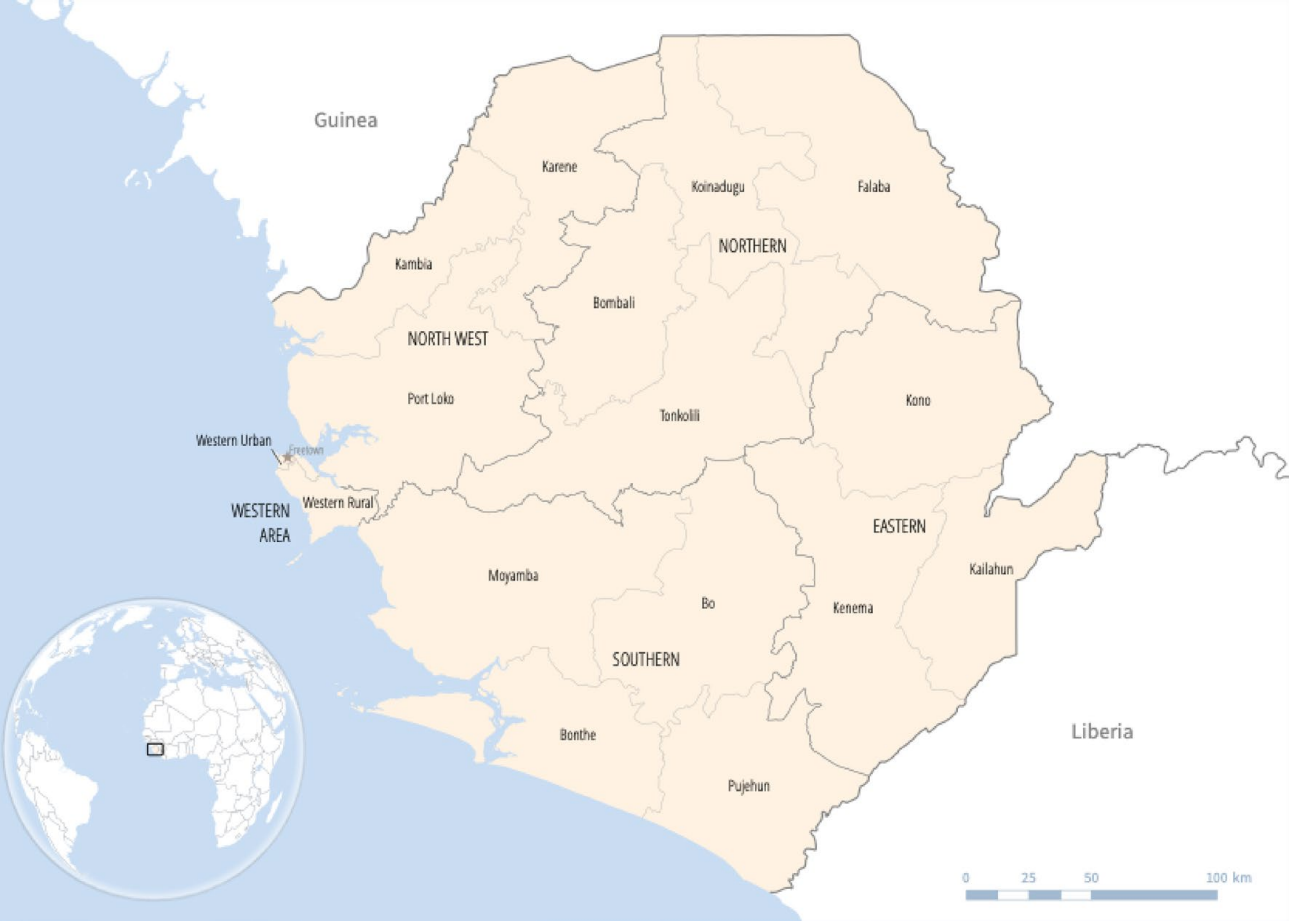
Map of Sierra Leone (Adapted from the Sierra Leone Demographic and Health Survey, 2019 [3])

Sierra Leone is a country on the coast of West Africa, bordered by Guinea and Liberia, with an estimated population of 7.9 million as of 2019, with 41.6% being under the age of 15 years and 59% residing in rural areas [1]. The life expectancy at birth for 2015–2020 is estimated to be 48.3 and 50.8 years, respectively, for males and females [1]. The country is divided into five provinces (Northern, Eastern, Southern, North West, Western Area (which contains the capital Freetown)) [2] (Fig. 1). The provinces are divided into 16 districts, further divided into 190 chiefdoms [2].

Sierra Leone is one of the countries with low human development and gender inequality indices [4]. For example, the country’s human development index is 0.477, positioning it at 181 out of 191 countries and territories, while the gender inequality index (GII) is 0.633, ranking it 162 out of 170 countries based on 2021 data [4]. Development and economic growth have been hampered by a decade-long civil war (1991–2002) and emergencies such as the Ebola outbreak (2014–2016) and the Freetown landslides (August 2017). Sierra Leone has had 16 disasters, most caused or exacerbated by human influences and resulting in injuries, deaths, and property losses [5]. Most of these have been caused or exacerbated by human influences, resulting in injuries, deaths, property losses and damages, and interrupted daily life support services, all of which have seriously affected the country’s growth and development [5]. These challenges are compounded further by an ongoing 2% population growth and high poverty level [6]. Estimated poverty rates are above 50%, and the estimated GDP growth for 2022 is nearly 3%, marking a reversal of the encouraging rebound observed in 2021 when GDP grew by 4.1% following a 2% contraction in 2020 [7].

Sierra Leone remains a country with high mortality and fertility rates and significant morbidity from preventable causes like malaria and malnutrition. For instance, the maternal mortality ratio (MMR) per 100,000 live births is estimated at 443; the under-five mortality rate per 1,000 live births is 105, and the total fertility rate is estimated at 4.2 [3, 8, 9]. There are relatively low health service coverage rates and high out-of-pocket (OOP) health expenditures. In 2020, OOP expenditure as a share of current health expenditure for Sierra Leone was 55.7% [10]. Though Sierra Leone's OOP expenditure as a share of current health expenditures fluctuated substantially in recent years, it tended to decrease through the 2001–2020 period, ending at 55.7% in 2020 [10]. Health services in the country are mostly delivered through a public system at three levels (with extended support provided by community health workers (CHWs) and district hospitals and referral hospitals providing secondary and tertiary patient care). This network of more than 1,200 health facilities includes 40 hospitals, of which the government operates 23, with the remaining owned by private entities and non-governmental and faith-based organisations. The health system is served by roughly 20,000 health workers of different cadres (this does not include CHWs or traditional birth attendants) [11]. Of these, some 9,000 are volunteers who are not on the government payroll, and there are roughly four medical doctors per 100,000 inhabitants [11]. Since 2010, health services have been free for pregnant women, lactating mothers, and children under five years [12–14]. Ebola survivors and people with albinism were later added to the free healthcare category. The country's health systems have many challenges, especially leadership and governance, financing, monitoring and evaluation, human resources and drugs, supplies and equipment [2].

Sierra Leone has an average Global Health Security Index (GHSI) of 32.7, essentially unchanged from 2019, showing continued weaknesses in global health security [15]. It has been shown that climate change, environmental degradation, poverty and governance challenges have mainly contributed to disasters in the country [5]. These factors have contributed towards more reactive disaster management in the country (focused on responding) rather than proactive (focused on preparedness—avoiding, preventing and mitigating disaster impacts). Furthermore, it has been shown that the politicisation of national issues has challenged disaster management, such as spreading fake news and rumours for political advantage during public catastrophes or providing contradictory actions and statements by state officials during disaster emergencies that create doubts in the victims [5].

Besides, disaster management has previously been hindered by the conflicting mandates among institutions with a role in disaster management [16]. For instance, state institutions such as the National Protected Area Authority and the Forestry Department are mandated to preserve Freetown’s hills as natural assets. At the same time, the Ministry of Lands is sometimes interested in awarding such lands for estate development as part of its land protection, planning and allocation mandates. This conflict in institutional mandate can sometimes affect land conservation and other disaster mitigation interventions. In all these, marginalised individuals and communities bear the brunt of the consequences [5, 16].

### State of disaster management and the evaluation gap

A disaster is a sudden catastrophic event that brings great damage, loss, or destruction [17]. Often, it disrupts a country’s well-being, safety, and functioning at any spatial level. This disruption is brought about by dangerous events interacting with insecurity, capacity, and exposure, leading to losses in lives, property, and environmental resources that may require external humanitarian assistance [5]. In medicine, a disaster is a ‘disruptive event whose destructive impact overwhelms a community’s ability to meet healthcare demands’ [17]. A disaster sometimes involves many human casualties, often referred to as mass casualty incidents (MCIs)). The number of injured patients overwhelms the capacity of local resources including at treating facilities. Especially in low- and middle-income countries, just a few injured patients can overwhelm an already stressed healthcare system.

Having learned from managing disasters in Sierra Leone, significant improvements have been achieved in emergency coordination and communication. For instance, the country's emergency governance structure has improved through forming institutions, such as the National Disaster Management Agency (NDMA), that have been established to forecast and manage disasters. Also, disaster communication is coordinated through the Ministry of Information in press conferences where all the agencies involved are represented. Besides, there have been other increased efforts to enhance disaster management through relevant assessments. For example, the Ministry of Lands, Country Planning and the Environment and the Freetown City Council evaluate natural disasters and risks in Freetown to improve the city's disaster mitigation [5]. Also, the country has developed elements such as a national risk map, disaster preparedness baseline, etc. Additionally, researchers have evaluated different risks, hazards, and disasters and their effects on the country's long-term economic sustainability [5]. Others have examined the national state-of-the-art disaster management in the country and shown the implementation drawbacks, research gaps, advances, and prospects [5].

Also, the Government of Sierra Leone identified several strategic priorities to address key institutional gaps to build a stronger, more resilient health system and expand access to affordable healthcare building on the Presidential Recovery Plan launched in mid-2015 [18]. Moreover, building on the Health Sector Recovery Plan (HSRP), the country is addressing priorities (targeting patients and health workers' safety, essential health services, health workforce development, community ownership, and information and surveillance) to build a stronger, more resilient health system, and expand access to affordable healthcare.

While there has been some progress from disasters and crises that the country faces, it is imperative to give a detailed account of the happenings of each disaster and learnings. Previously, the country attempted to evaluate its response to the Ebola Crisis in 2014–2016, which was useful in remodelling its emergency response. However, there has been a gap in having similar reflections on the management of the other responses that the country has faced. Therefore, this study aims to build emergency response learnings by reflecting on Sierra Leone's experience managing the fire incident following the tanker explosion and the international and national emergency medical teams (EMTs) role in response to the incident.

## Method

**Fig. 2:**
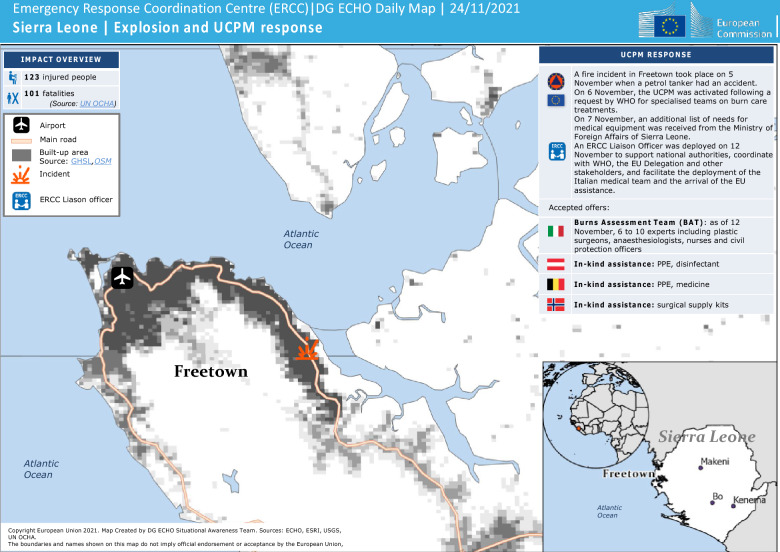
Map of the explosion site and some EU Civil Protection Mechanism (UCPM) responses (Source: Sierra Leone | Explosion and UCPM response | 24/11/2021 [20])

This study is a cross-sectional After-Action Review (AAR) debrief of the response to a recent mass burn incident following a tanker explosion in the Wellington PMB Junction east of Freetown, Sierra Leone. Using the WHO guidance for after-action review (AAR) to review actions taken in response to public health events [19], the WHO (HQ, AFRO, and Country Office) conducted an after-action review of the EMT response to the explosions in Sierra Leone to generate knowledge about strengths and opportunities for improvement to inform future responses. The process consisted of three stages: document review, a debrief, and analysis. The document reviews included mission reports, assessments, meeting minutes on coordination, information from key websites of WHO and MoH, and media coverage (local and international). The debrief was a virtual facilitator-led discussion comprising WHO and MoH staff (n = 14) held over two hours after the response. The small group permitted a diverse discussion on the response around EMT mobilisation, deployment, coordination, case management activities, national capacity and community acceptance. The scope of the discussion was to allow focused learning of the thematic areas around the event and the responses of different teams in addition to the review of EMTs' responses. Analysis was done thematically following into the identified areas (Fig. 2).

## Results

### The event and the initial response

On 5 November 2021, a road traffic accident that resulted in a fire incident occurred in the Wellington PMB Junction east of Freetown. It involved a loaded fuel tanker colliding with a truck carrying granite stones while the fuel tanker was about to enter a nearby filling station to discharge its fuel. It was reported that the granite lorry lost its brakes and ran into the tanker, which led to fuel spillage from the tanker. Nearby bike riders and traders rushed to the scene for free fuel. Further, some community members rushed to the scene and took advantage of the leakage to scoop fuel and store it in nearby makeshift structures. This rampage lasted for about 45 min before the explosion and resulted in an inferno, killing nearly 101 people and injuring about 123 [21, 22]. Both public and private properties such as vehicles, gas stations, shops and dwelling houses got burnt, and there was distress and trauma to families of those affected [22].

#### The arrival of government agencies at the scene and initial coordination

Immediately after the explosion, officials from the then-newly formed National Disaster Management Agency (NDMA) arrived at the site. They established an early ad-hoc coordination mechanism to coordinate the fire service, National Emergency Medical Service (NEMS) ambulances, and the police to cordon off the area.

#### Management of the victims

Many fire victims were transferred to various hospitals within the township, including Connaught (the national referral hospital), 34 Military, the Rokupa Government Hospital, Choithram Memorial Hospital (a private hospital), and Emergency Surgical Center (an NGO). The deceased, in the hospitals that they were tranferred to, were preserved in the same hospitals; however, initially, there was a challenge with the identification of victims by relatives due to the extent of the burns. Connaught Hospital initially became overwhelmed, but the other hospitals supported handling the patients. Patients were triaged, treated or admitted as suggested by the hospital’s burns case management team (of plastic surgeons, general surgeons, anaesthesiologists, physicians, nutritionists and nurses).

On the first night of the disaster, Connaught Hospital received 82 severely injured patients and 87 corpses of burnt persons, with three doctors and eight nurses as the ground staff on duty at the accident and emergency department (A&E). In the first line of action, the hospital repurposed nursing staff from other wards to the A&E department and those not on duty were called to support the overwhelming patient load. The hospital store and pharmacy used their available supplies and later received supplies of drugs and consumables from the MoH central medical stores. Triage and separation of patients based on the degree of burns and the chance of survival were done by the staff in the accident and emergency (A&E), and others were transferred to the other existing hospital wards for initial resuscitation of the burns patient.

Despite these measures, it became evident that the scale of the situation exceeded the country's existing capacity for trauma/burns management. The overwhelming nature of the crisis exposed the limitations in fully addressing the growing demands and complexities. Additional support and resources were required to bolster response efforts.

As of 30 November 2021, 304 people had been affected by the incident, with 35 admitted to 5 city hospitals [23]. Additionally, there was a destruction of property, including vehicles and business premises (2 residences of 5 households, 22 vehicles, 48 motorbikes, and three tricycles); a disruption of the social and economic life of households within one kilometre from the site (catchment population approx. 35,605); destruction of vegetation in the surrounding environment and air pollution [23].

#### Establishment of the incident management team (IMT) and informing partners

Immediately, through MoH, the government convened the National Public Health Emergency Management Committee (PHEMC) on 6 November to inform partners of the incident and their initial actions so far. Following the initial Emergency Operation Center (EOC) meeting, another meeting was held on 6 November to inform partners of the assessed damage further and to set up the incident management team to coordinate the response. Implementing Incident management principles outlined in the Emergency Response Framework [24], such as timely assessment and event grading, facilitated the timely deployment of critical resources.

Afterwards, the PHEMC activated the Emergency Operation Center (EOC) Level 2 incidence response [22]. An Incident Management Team (IMT) was appointed by the Hon. Minister of Health and the leadership of the MoH to coordinate the medical response. The government informed partners that over 100 burn patients had been transported to various hospitals across Freetown, and medical practitioners were being pulled from their locations to support case management in these hospitals. In the meantime, the IMT had activated all the pillars, including case management, laboratory, logistics, infection prevention and control (IPC), Risk Communication and Community Engagement (RCCE), and surveillance. The established IMT coordinated activities in all hospitals where patients had been taken, including Connaught Hospital. The IMT established an interdisciplinary team comprising plastic surgeons, general surgeons, orthopaedic surgeons, anaesthetists, physicians, nurses, physiotherapists, and nutritionists who used the standardised management plans established under the IMT. The Minister of Health also authorised the MoH team to use the medical equipment and commodities in stock to respond to the incident and requested all partners to support the cause. During the IMT meeting on 6 November 2021, Médecins sans Frontières (MSF) committed to providing medical equipment and commodities to support case management at different hospitals [22].

#### Support from the EMTs

**Fig. 3:**
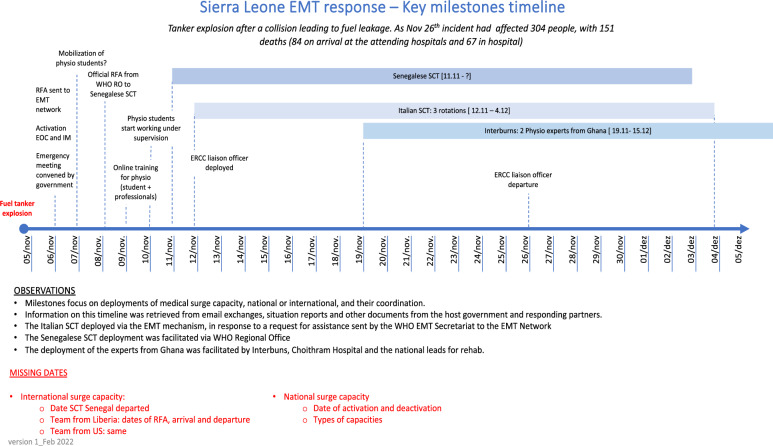
Timelines of activities

A summary of the timeline of the EMT activities is in Fig. 3. The government requested support from EMTs (surge teams) from Senegal, the United States of America, Liberia, Italy and China to support the emergency response to the disaster. WHO conducted a Rapid Risk Assessment and an independent grading, quickly accessing financial resources through the Central Fund for Emergencies (CFE) to expedite the initiation of emergency response interventions. WHO provided six tons of critical case management commodities and supplies within 24 h of notification and deployed a specialist emergency medical team (EMT) comprising an orthopaedic/trauma surgeon, anesthesiologist, visceral surgeon, and several specialist nurses [25]. In addition to supplementing the work of local experts, visiting specialists also provided training to help strengthen local staff capacity. A roster approach to deploying Emergency Medical Teams and other experts facilitated a further swift response. On 6 November, WHO requested specialised care teams (SCT) on burns care treatments, and on 7 November, an additional list of needs for medical equipment was received from the Ministry of Foreign Affairs of Sierra Leone [20].

The role of the WHO AFRO in identifying, mobilising and deploying the team from Senegal was key. The process was based on the success of their previous deployment in DRC to manage a fire incident two years prior. The WHO AFRO supported them financially and technically in the mobilisation and ensured that medicines and consumables for the response were available. Besides, the WHO AFRO facilitated the interaction with the WHO Country office (WCO) of Sierra Leone. The deployment was done within 72 h. Furthermore, there was an opportunity to pre-test and validate the personnel deployment plan that MoH developed. Within record time, briefing was done for all relevant stakeholders on the role of the Senegalese EMT. They obtained licenses to practice before providing any healthcare services to the burn victims.

The government accepted offers from the Italy Burns Specialized Team (BST), and by 12 November, 6 to 10 experts, including plastic surgeons, anesthesiologists, nurses, and civil protection officers, had arrived. Others offered in-kind assistance. For instance, Austria offered PPE and disinfectant; Belgium offered PPE and medicine; and Norway offered surgical supply kits [20].

Daily coordination meetings effectively managed the activities of the local team and the surge teams. On-the-job training of local staff on burns care by experts (local and international) was also carried out. The disaster attracted donations from nongovernmental organisations (NGOs), Government partners at home and abroad, religious organisations and well-meaning Sierra Leoneans. These donations included food, water, intravenous fluids, drugs and other items. In collaboration with the National Medical Supplies Agency, the hospitals set up a system to ensure that the donated items were evenly distributed among the hospitals caring for burn patients.

### Review of the EMT response

#### Mobilisation and deployment of the EMT

WHO demonstrated leadership in responding to the incident. The WHO AFRO regional team led the guidance and support of the deployment process. The regional office requested the deployment of the Senegalese International EMT and mobilised resources, including medical supplies, drugs and other consumables, which were shipped from the Ghana warehouse, and these were cleared from the Sierra Leone ports and delivered to the ministry by ministry officials. The first batch of one metric ton of the kits was delivered on 7 November, while the remaining 5.6 metric tons were successfully delivered to the Ministry of Health and Sanitation a day later. The emergency medical kits contained medicines, fluid infusions, disinfectants, autoclave sterilizer, dressings for burns and gloves. These commodities were estimated to treat as many as 600 people with severe burns [26].

Based on a previous similar experience in DRC in 2018, the Senegalese EMT was mobilised and deployed 72 h after the explosion of the tank. The collaboration, support, and actions of the WHO County Office and the Senegalese Medical Military authority were timely. The Sierra Leone WCO team facilitated the receipt of the Senegalese team and, in sync with the IM team in the ministry, provided accommodations and other logistics support to ensure the EMT team was comfortably settled in to provide the required services.

#### Coordination

The coordination was a three-level coordination by HQ, AFRO, and the in-country team (WCO and MOH) and was properly instituted. WHO also supported the country through expert advice and provided initial funds from the Contingency Funds for Emergencies to facilitate operations activities on the ground. Upon arrival, the EMT members were briefed on the situation by the WCO case management team lead and the IMT at the Ministry of Health. After the team submitted a work roster, they were posted to the three hospitals where the patients were admitted. Of significance was the foresight by the team to bring along equipment to support their work whilst they waited for clearance and handover of the supplies shipped by WHO and other partners, which were sent directly to the EMT at Connaught and were stored in the surgery dept for use to do surgeries, wound dressings, including consumables, drugs (antibiotics, analgesics, anaesthesia agents etc.). There may have been accountability issues for the supplies provided, but the WHO team constantly interacted with colleagues in MoH as this provided a basis for informal engagements and reduced the bureaucratic processes involved in setting up meetings with MoH and partners.

The National Emergency Medical Services (NEMS) team and a disaster response unit were notified following the incident; however, the MoH may not have harmonised the coordination well, leading to gaps in the triage and transport of patients to the facilities. Nonetheless, the patients were transported via buses, motorbikes, taxis and private vehicles. This prompted the call for the government to build the capacity of the NEMS and strengthen collaboration amongst the various health partners. As summary of the numbers is shown in Table 1.

**Table 1:** A summary of the number of patients and deaths. Source: Ministry of Health

Hospital	Total patients received	Total patients discharged	Total deaths in hospital	Total deaths at scene
Connaught	87	59	27	
34 Military	26	10	16	
Emergency	27	13	17	
Rokupa	9	9	0	
Choithram	8	3	5	
Total	157	94	65	86

However, while there were some constraints with the coordination processes, a dedicated team at the MoH Command was responsible for accelerating the process of registration and licensing of the EMT members with the Sierra Leone Medical and Dental Council (SLMDC). This was aimed at securing practising licenses to enable teamwork in the country, which was part of the legal requirement and regulations by the SLMDC that all healthcare professionals must be licensed and registered by the accredited body before the postings/ deployment of healthcare workers to facilities to provide health care. While this contributed to better timeliness of the response to provide care to the patients, it led to the suggestion that in future, the credentials/ documents of EMT members should be forwarded (immediately) after they are contacted for deployment whilst other preparatory procedures, such as travel arrangements, are ongoing to hasten the response further.

#### Case management activities

The EMTs were instrumental in providing burns and psychosocial care to all the patients admitted to the three health facilities. The clinical care provided included reconstructive surgery for some patients, pain management and palliative care, wound dressing, rehabilitation and physiotherapy, psychosocial counselling, and daily debriefing activities of the cases managed. The clinical expertise and the case management services offered by the EMTs work helped to reduce the deaths and post-burn sequelae. Further, the EMTs provided other clinical teams with on-the-job training in burns management and palliative care. The daily technical update provided by the team was vital not only in knowledge dissemination but also in acquiring information that was used to provide briefings to senior management of the MoH and the Public Health Emergency unit.

However, there were immense gaps in managing such incidents. The country lacked the capacity for mass casualty management and had few general surgeons, only two reconstructive surgeons, one trauma surgeon, and few emergency and critical care nurses. Equally, the team encountered challenges in managing the patients' prime: the unavailability of a burns unit, and patients had to be treated either in the ICUs or makeshift HDUs.

As such, it led to deliberations with partners to erect a burns unit, as burns patients are highly susceptible to nosocomial infections. This request has been made, and the government is working with the private sector to build a burns unit at Connaught. Construction has already started. Furthermore, there was a gap in the availability of validated standard operation procedures (SOPs) for use as a crucial element to improve patient safety and quality of care, and the EMT owned most of the equipment used during this period. These actions led to the hastening of the ongoing process of having the MoH participants set up a national EMT, and West African Health Organization (WAHO) started it by conducting a stakeholder engagement.

#### Available national capacity and community acceptance

During this period of the tanker explosion, the national capacity, including health workers, medical supplies and logistics to respond to the incident, was low, and there was a shortage of responders to public health emergencies. The EMT deployment was critical and augmented the available human resources to provide the much-needed clinical care.

The EMTs dispensed their clinical functions with high professionalism and were quite flexible in adapting to the situation and the resources available, and this was attributed to similar backgrounds and culture, the team having emanated from Senegal, an LMIC in the West African Coast. Further, the language barrier, perceived as a weakness in providing care and a barrier in communicating with patients, did not hinder the patient-doctor/ nurse relationship or the provision of clinical care. Towards the end of the deployment period, it was noted that the Senegalese EMT had built a strong relationship with the patients and other healthcare workers, caregivers, families and well-wishers in the communities. By the end of the deployment period, the national clinicians benefitted from some skills acquisition. This knowledge transfer empowered local staff to assist victims in conjunction with the Emergency Medical Team. This was pivotal in substantially increasing the survival rates of severely injured people being treated in hospitals suggesting to adopt a systematic approach for strengthening local burns care capability. Further, the community members appreciated the team's work; of the 157 patients admitted to the five facilities, 94 were discharged home.

## Conclusion

In this study, we documented Sierra Leone's experience in managing the fire incident following a tanker explosion and the international and national EMTs' role in responding to it. We have highlighted that the collaboration between the MoH and NDMA was imperative in instituting a well-coordinated response mechanism that managed all response actions, such as medical services, informing partners and the public and coordinating all other agencies. WHO facilitated EMT deployments and mobilised medical supplies and equipment. The EMTs dispensed their functions with professionalism, adapted to the environment and available resources, and augmented the care the national health workers provided. Despite being perceived as a weakness, the language barrier did not hinder the patient-doctor/ nurse relationship or the provision of clinical care. The joint work between the government, partners and EMTs was important in the response. It enhanced the response speed, political acceptability and health context experience to support rapid and safe deployment. This experience brought to focus the idea of developing a national EMT in Sierra Leone, which would be useful to help respond swiftly to disasters in the Mano River basin.

## Recommendation

There is a need to institute further mechanisms to facilitate rapid response and quality-assured deployment of EMTs at regional and sub-regional levels in collaboration with partners such as WHO, which should be strengthened in the region to support future responses. For instance, the country needs to:

1. Strengthen coordination among partners with better provision, allocation and use of resources (both donor and government).
2. Build the emergency response capacity of HCWs through establishment of national EMT program to support disasters locally and internationally.
3. Establish a burns unit at a national hospital with support from WHO and other partners.
4. Establish an EMT in all tertiary hospitals to intra-hospital emergencies and when needed, provide surge support to the health system in response to mass casualty incidents, including strengthening local burns care capability and providing mass casualty management for first-line responders with the support of WHO.
5. Develop recovery plans for victims, including compensation, restoration of livelihood for survivors and their dependents, and healthcare delivery system improvement.

## Data Availability

All data used in this study are part of the manuscript.
